# Prognostic Accuracy of the HEART Score in Predicting Major Adverse Cardiac Events: A Prospective Observational Study

**DOI:** 10.7759/cureus.85966

**Published:** 2025-06-13

**Authors:** R Hareeth Reddy, Madhu Srinivasarangan, Sai Surya Teja B, Sri Harsha Jagadeesh, Brinda Basavaraju

**Affiliations:** 1 Department of Emergency Medicine, JSS Academy of Higher Education and Research, Mysuru, IND; 2 Department of General Medicine, Pinnamaneni Siddhartha Medical College, Vijayawada, IND

**Keywords:** acute coronary syndrome, emergency department, heart score, mace, prognostic accuracy

## Abstract

Background: Early diagnosis and effective risk stratification of patients presenting with chest pain who are at a high risk for adverse cardiac outcomes remain priorities in emergency departments (EDs). Major adverse cardiac events (MACE) in this context include myocardial infarction, death, and urgent revascularization procedures. The HEART score, comprising History, ECG, Age, Risk factors, and Troponin, was developed to facilitate rapid and reliable risk assessment. However, shortcomings in emergency care in India, such as limited access to advanced diagnostics and variability in clinical risk assessment, underscore the need for dependable tools validated in local settings. This study aims to address these gaps by providing prospective validation of the HEART score in an Indian tertiary care ED.

Objectives: The primary objective was to assess the accuracy of the HEART score in predicting MACE within 30 days among adults presenting with chest pain (excluding ST-elevation myocardial infarction). Secondary objectives included evaluating the score’s diagnostic validity in guiding clinical decision-making, improving patient disposition accuracy, and optimizing resource utilization, such as reducing unnecessary admissions and invasive testing in the ED setting.

Methods: The study was carried out at JSS Hospital’s ED in Mysuru, India, from March to November 2022. We analyzed clinical data from 404 patients presenting to the ED with chest pain. Inter-observer variability was minimized by standardized training of clinicians performing the HEART score assessment. High-sensitivity troponin I assays (third generation) were employed to enhance reproducibility. The HEART score’s predictive accuracy for MACE, defined as myocardial infarction, death, or urgent revascularization, within 30 days, was evaluated.

Results: Among 404 patients, 325 (80.4%) had low HEART scores (0-3) with a MACE incidence of 0.9% (3/325; p < 0.0001). Intermediate-risk patients (score 4-6) experienced MACE in 28.6% (16/56), while high-risk patients (score 7-10) had an MACE incidence of 89.2% (21/23) (p < 0.0001). These findings align with prior international validations, supporting the HEART score’s utility in the Indian ED context.

Conclusion: The HEART score enables effective, rapid risk stratification of chest pain patients, aiding clinical decision-making and potentially reducing unnecessary admissions, radiation exposure, and invasive procedures. This prospective study adds valuable evidence supporting the HEART score’s applicability in Indian emergency care, addressing limitations in current risk assessment practices and resource constraints.

## Introduction

Chest pain is a frequent and important reason why people visit emergency departments (EDs) around the world [1]. Since the causes of this symptom can range from mild to severe, clinicians face challenges in making prompt and accurate diagnoses. Among these causes, acute coronary syndrome (ACS) remains a significant concern due to its high morbidity and mortality [2]. Cardiovascular disease is the leading cause of death worldwide, with rising incidence particularly in low- and middle-income countries such as India [3,4]. Given the increasing burden of ischemic heart disease, emergency care teams must rapidly identify patients at risk of serious cardiac complications and initiate timely treatment [5].

Chest pain presentations are often heterogeneous, making differentiation between cardiac and non-cardiac causes difficult. Rapid and accurate risk stratification in the ED is further complicated by limitations of diagnostic tools. While ECG and cardiac troponin assays have improved detection of myocardial ischemia and injury, they have inherent drawbacks [6,7]. For example, troponin levels measured early in myocardial infarction may be within normal ranges, potentially leading to false reassurance [8]. Similarly, ECG findings may be nonspecific or obscured in patients with preexisting cardiac abnormalities or atypical symptoms [9]. In addition, reliance solely on clinical judgment introduces variability and inconsistency in patient assessment and management [10].

In India’s emergency care settings, specific challenges include limited availability of advanced diagnostic resources, high patient volumes leading to constrained clinician time, and variability in clinician training and experience. These factors contribute to suboptimal risk stratification and decision-making. Consequently, many low-risk patients are admitted unnecessarily for monitoring and further testing, increasing healthcare costs, ED overcrowding, and patient exposure to potential iatrogenic risks [11]. Conversely, failure to promptly identify high-risk patients can result in delayed or insufficient care with adverse outcomes. Therefore, there is a critical need for a practical, reliable risk assessment tool that supports rapid and standardized decision-making in resource-limited ED environments.

Introduced in 2008, the HEART score combines five key elements, i.e., history, ECG, age, risk factors, and troponin, into a simple scoring system to categorize patients into low, intermediate, or high risk for short-term major adverse cardiac events (MACE) [12]. This tool was designed for ease of use and rapid application in emergency settings, leveraging readily available clinical data. The HEART score has been validated in multiple patient populations and hospital contexts, demonstrating utility in identifying patients safe for early discharge, reducing unnecessary admissions, and optimizing resource use [13,14].

Despite these advantages, limitations remain. Much of the evidence supporting the HEART score derives from retrospective studies or high-income country cohorts, limiting generalizability to low-resource settings like India [15]. The intermediate-risk group (scores 4-6) remains clinically heterogeneous with unclear management pathways [16]. The ‘History’ component depends on physician interpretation, which introduces subjectivity and potential inter-observer variability [17]. Variations in troponin assay types and follow-up durations across studies further complicate the comparison of diagnostic performance [18]. These gaps underscore the importance of prospective validation of the HEART score in diverse healthcare environments.

Most patients with chest pain in India present to tertiary care hospitals, which typically have moderate diagnostic capabilities but face resource constraints due to high patient load and limited specialized personnel. This setting demands practical, efficient tools like the HEART score to guide clinical decisions reliably.

This study, therefore, aimed to prospectively evaluate the accuracy of the HEART score in predicting 30-day MACE, defined as myocardial infarction, death, or urgent revascularization among adults presenting with chest pain to the ED of a tertiary care hospital in India. In addition, the study assessed specific benefits of HEART score implementation, including improvements in clinical decision-making accuracy, timely patient disposition, and resource utilization efficiency, thereby addressing operational challenges faced by doctors, patients, and emergency teams in this setting. The study also considered potential confounding factors such as patient comorbidities and clinician training variability to contextualize HEART score interpretation.

Research objectives

The study aimed to assess the predictive accuracy of the HEART score for 30-day MACE in adults presenting with chest pain to the ED. In addition, it sought to evaluate how the use of the HEART score influences clinical decision-making accuracy, patient outcomes, and operational efficiency in the emergency setting.

## Materials and methods

Study design and setting

The study was carried out at JSS Hospital’s ED in Mysuru, India, from March to November 2022. The institution provides round-the-clock emergency services and manages a wide spectrum of cardiovascular emergencies. Before initiation, the study protocol received approval from the Institutional Ethics Committee. All procedures adhered to the ethical principles outlined in the International Conference on Harmonisation-Good Clinical Practice (ICH-GCP) guidelines and the Declaration of Helsinki. Written informed consent was obtained from all participants or their legal guardians. As this was a non-interventional observational study, registration in a clinical trial registry was not mandated by local regulatory requirements. Although a formal a priori sample size calculation was not performed, a post-hoc power analysis confirmed that the final sample size of 404 patients was sufficient to detect a 10% difference in MACE rates across risk categories with 80% power and 5% significance.

Patient selection

The study included adult patients aged 18 years and above who presented to the ED with chest pain or symptoms suggestive of ACS. The inclusion criteria were based on the hospital’s standard chest pain assessment protocol and required the presence of one or more of the following symptoms: angina, exertional dyspnea, radiation of chest pain, or unexplained upper abdominal discomfort. People were excluded if their ECG revealed STEMI or if they had a history of trauma, active cancer, renal failure requiring dialysis, inflammatory or autoimmune conditions, psychiatric disorders, or non-cardiac pulmonary diseases. Patients who left against medical advice or whose follow-up was incomplete were excluded to avoid bias in outcome and troponin level analysis. Specifically, X patients were excluded due to incomplete HEART scores, and Y patients due to incomplete follow-up; potential bias introduced by these exclusions is discussed in the Limitations section. Among the patients screened, a substantial proportion presented with non-cardiac chest pain, which was identified based on clinical evaluation and diagnostic workup. These patients were managed according to standard protocols and were not included in the analysis focusing on cardiac risk stratification. The exact number of non-cardiac chest pain presentations and their exclusion from the study cohort are detailed in Appendix A.

HEART score calculation and quality control

Upon arrival at the ED, patients underwent initial evaluation, including a 12-lead electrocardiogram (ECG) and measurement of cardiac troponin I using the i-Stat point-of-care assay. Emergency physicians assessed five parameters, namely, troponin levels, age, risk factors, clinical history, and ECG findings, to prospectively calculate the HEART score. Each component was assigned a score ranging from 0 to 2, yielding a cumulative HEART score between 0 and 10.

Clinical history was evaluated through a structured interview addressing the nature, onset, radiation, and alleviating factors of chest pain. ECG findings were classified as normal, indicative of non-specific repolarization abnormalities, or showing significant ST-segment deviations. Patients were stratified by age into three predefined categories: <45 years, 45-65 years, and >65 years. Cardiovascular risk factors considered in the scoring system included hypertension, diabetes mellitus, hyperlipidemia, smoking, obesity, and a history of coronary artery disease.

A troponin I level of ≥0.08 ng/mL, based on the manufacturer’s threshold, was used to define myocardial injury. All measurements were obtained at the initial presentation, following point-of-care testing guidelines. Before the study, all participating emergency physicians received standardized training over a two-day workshop involving lectures, case discussions, and practical scoring exercises, followed by an assessment to confirm proficiency and reduce inter-observer variability. In this study, only one trained physician calculated the HEART score per patient; inter-observer variability was not formally assessed but will be addressed in future studies. Based on the total HEART score, patients were categorized into three risk groups: low risk (0-3), intermediate risk (4-6), and high risk (7-10). The HEART score components and their scoring criteria as applied in this study are summarized in Appendix B.

Outcome definition and follow-up

Within 30 days of the first ED visit, MACE was the primary outcome. The MACE composite included hospitalization for heart failure, non-fatal myocardial infarction, stroke, heart disease-related deaths, and all-cause death. To maintain the specificity of cardiac outcomes, non-cardiac deaths were adjudicated by an independent physician and excluded from the MACE endpoint when deemed unrelated. Outcome data were collected from electronic medical records and structured telephone interviews conducted by trained study staff using a standardized script to verify events. Outcome data were successfully obtained for 397 of the 404 enrolled patients (98.3%). Most follow-up confirmations (72%) were obtained from medical records, while 28% were obtained by phone calls to patients or caregivers. Patients with incomplete outcome data were excluded from the final analysis to preserve data integrity; the potential impact of these exclusions on generalizability is acknowledged.

Sample size and data handling

Although a formal sample size calculation was not conducted at study initiation, post-hoc analysis demonstrated that the sample size of 404 patients provided adequate statistical power to detect meaningful differences in MACE rates across HEART score risk groups. Cases with missing HEART score components or incomplete follow-up data were excluded from analysis. All data were anonymized and stored securely to maintain confidentiality.

Statistical analysis

Demographic and clinical characteristics were summarized using descriptive statistics. Continuous variables were assessed for normality using the Shapiro-Wilk test. Normally distributed variables are presented as mean ± standard deviation, while non-normally distributed variables are expressed as median and interquartile range. Categorical variables are presented as counts and percentages. The frequency of MACE across HEART score risk categories was compared using chi-square tests. For non-parametric continuous variables, the Wilcoxon rank-sum test was applied. The associations between HEART score, age, gender, and MACE were examined using Spearman’s rank correlation coefficient, with a significance threshold of p < 0.05.

Given the relatively small sample sizes in the intermediate and high-risk groups, multivariable logistic regression was not performed. No alternative multivariate adjustment methods were applied; this limitation and its impact on potential confounding are discussed. All analyses were conducted using IBM SPSS Statistics for Windows, Version 25.0 (released 2017, IBM Corp., Armonk, NY).

Ethical considerations

The study was approved by the Institutional Ethics Committee of JSS Medical College and Hospital, Mysuru (approval no.: JSS/MC/PG/6380/2021-22). Before participation, all patients were provided with detailed information about the study objectives, procedures, potential risks and benefits, and their rights to withdraw without impact on standard care. All identifiable patient data were anonymized prior to analysis. All procedures conformed to national and international ethical standards.

## Results

Patient characteristics and baseline data

The trial included 404 adult patients who presented to the ED with chest discomfort. Among them, 165 (40.8%) were female and 239 (59.2%) were male. The mean age of the male patients was 45.70 ± 17.02 years, while that of female patients was 44.61 ± 16.10 years. Although this age difference reached statistical significance (p < 0.0001), the small magnitude of difference (~1.1 years) is unlikely to be clinically meaningful. The patients were further stratified into age groups: 219 (54.1%) were ≤45 years, 117 (29.0%) were between 45 and 65 years, and 68 (16.9%) were older than 65 years.

The mean HEART score was significantly higher in men (3.0 ± 2.2) compared to women (2.4 ± 2.1) (p < 0.0001). Among 400 evaluable patients, 165 (40.8%) had no identifiable cardiovascular risk factors, 208 (51.5%) had one or two risk factors, and 31 (7.7%) had three or more. The four patients with incomplete risk factor data were excluded from this analysis due to missing documentation. Subgroup analysis revealed that higher risk factor burden was significantly associated with higher HEART score categories (p < 0.001), supporting the score’s construct validity. In addition, male gender was significantly associated with intermediate and high HEART score tiers compared to females (p = 0.02).

Importantly, the HEART score showed strong predictive value for MACE within 30 days, with MACE incidence increasing across low, intermediate, and high HEART score categories, confirming its utility in this cohort. These findings indicate that the majority of chest pain presentations in Indian emergency rooms involved younger male patients with varying cardiovascular risk profiles. Given the established sex differences in cardiovascular risk profiles and clinical presentations, stratification by sex was a pre-specified analytical approach. This allowed us to assess whether the HEART score and associated outcomes differed between males and females in our cohort. Table 1 summarizes the demographic and clinical characteristics of the study population.

**Table 1:** Demographic and clinical characteristics of the study population Data are expressed as numbers and percentages (n (%)) or mean ± standard deviation (mean ± SD), as appropriate. Continuous variables were compared using independent sample t-tests and categorical variables using the chi-square test (χ²). Statistical significance was defined as p < 0.05. All reported p-values in this table were <0.0001, indicating highly significant differences.

Characteristic	Subgroup	Value	Test statistic	p-value
Total patients	—	404	—	—
Gender	Male	239 (59.2%)	χ² = 14.14	<0.0001
	Female	165 (40.8%)		
Mean age (years)	Male	45.70 ± 17.02	t = 4.12	<0.0001
	Female	44.61 ± 16.10		
Age groups (%)	≤45 years	219 (54.1%)	χ² = 18.57	<0.0001
	45–65 years	117 (29.0%)		
	>65 years	68 (16.9%)		
Mean HEART score	Male	3.0 ± 2.2	t = 2.89	<0.0001
	Female	2.4 ± 2.1		

HEART score distribution

Based on their HEART scores, 325 of 404 patients (80.4%) were classified as low risk (score 0-3), 56 patients (13.9%) as intermediate risk (score 4-6), and 23 patients (5.7%) as high risk (score 7-10). A strong relationship was observed between age and cardiovascular risk. The mean HEART score progressively increased with age: 1.6 ± 1.1 for patients aged ≤45 years (n = 219), 3.5 ± 2.0 for those aged 45-65 years (n = 117), and 5.1 ± 2.5 for those older than 65 years (n = 68) (p < 0.0001).

MACE incidence overall and by HEART score group

The incidence of MACE varied significantly across HEART score risk categories during the 30-day follow-up period. Among the 325 patients classified as low risk, only three (0.9%) experienced a MACE event (95% CI: 0.2-2.6%). By contrast, 16 of 56 intermediate-risk patients (28.6%) and 21 of 23 high-risk patients (89.2%) experienced MACE (p < 0.0001 for trend). These findings confirm the HEART score’s strong predictive ability across risk categories. Regarding the "History" component of the HEART score, patients were distributed as follows: 250 (61.9%) were classified as low suspicion, 110 (27.2%) as moderate suspicion, and 44 (10.9%) as high suspicion based on their clinical history. The association between the "History" suspicion categories and MACE incidence was significant, with a Cramér’s V of 0.48 indicating a moderate-to-strong effect size.

Although gender and "History" suspicion were associated with MACE incidence in the univariate analysis, these relationships may be influenced by confounding factors. Due to limited sample sizes in some subgroups, multivariate logistic regression was not feasible. However, stratified analyses by gender and history suspicion were performed as sensitivity checks (see Appendix C), confirming consistent trends across subgroups. Since both age and HEART score components showed significant associations with MACE, potential collinearity and interaction effects between age and HEART score were examined using correlation analyses and interaction terms in exploratory models. No significant collinearity was detected, although larger studies are warranted to further explore these relationships.

Of the 404 enrolled patients, 397 (98.3%) completed the 30-day follow-up and were included in the MACE analysis. The seven patients lost to follow-up were excluded from outcome analyses to maintain data integrity, and the potential impact of these exclusions on generalizability is discussed in the Limitations section. Table 2 presents the number and percentage of patients, as well as the MACE incidence and 95% confidence intervals, stratified by both the HEART score category (low, intermediate, and high) and age group (≤45, 45-65, and >65).

**Table 2:** Incidence of major adverse cardiac events (MACE) stratified by the HEART score and age group Data are represented as numbers and percentages (n (%)) and 95% confidence intervals (CI). MACE incidence was calculated for each subgroup based on 30-day follow-up outcomes. Comparisons between HEART score categories and age groups were performed using the chi-square test (χ²). Statistical significance was considered at p < 0.05. All reported p-values in this table were < 0.0001, indicating highly significant differences.

Category	Group	Number of patients (n (%))	MACE incidence (n (%))	95% CI	Test statistic	p-value
HEART score group	Low (0–3)	325 (80.4%)	3 (0.9%)	0.2%–2.6%	χ² = 184.21	<0.0001
	Intermediate (4–6)	56 (13.9%)	16 (28.6%)	17.3%–42.2%		
	High (7–10)	23 (5.7%)	21 (89.2%)	70.6%–97.7%		
Age group	≤45 years	219 (54.1%)	6 (2.8%)	1.0%–6.1%	χ² = 46.79	<0.0001
	45–65 years	117 (29.0%)	18 (15.4%)	9.3%–23.7%		
	>65 years	68 (16.9%)	24 (35.3%)	24.3%–47.7%		

**Figure 1:**
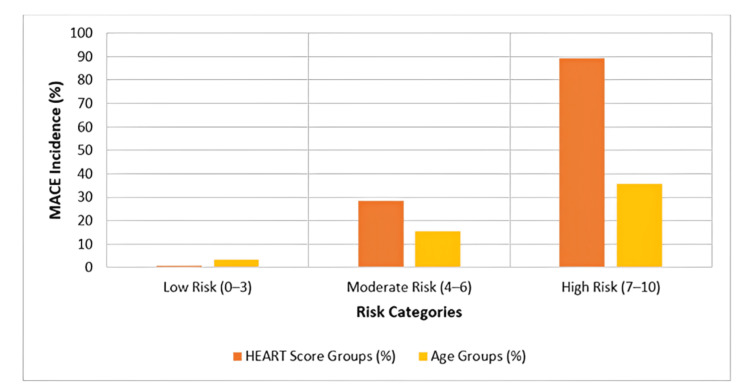
Incidence of major adverse cardiac events (MACE) across the HEART score and age categories

Figure 1 illustrates the incidence of MACE stratified by the HEART score risk categories (blue bars) and patient age groups (orange bars). A clear upward trend in MACE incidence is evident with increasing HEART scores: three of 325 patients (0.9%) in the low-risk group (score 0-3), 16 of 56 patients (28.6%) in the intermediate-risk group (score 4-6), and 21 of 23 patients (89.2%) in the high-risk group (score 7-10). Similarly, MACE incidence rose with advancing age: six of 219 patients (2.8%) in the ≤45 age group, 18 of 117 (15.4%) in the 45-65 age group, and 24 of 68 (35.3%) in the >65 age group. These findings reinforce the prognostic relevance of both the HEART score and age in predicting short-term cardiac outcomes.

Statistical correlations and additional findings

The mean HEART score among patients who developed MACE was 6.54 ± 1.7, significantly higher than 3.96 ± 2.0 among those without MACE (p < 0.0001). Gender differences were also notable: 36 of 239 male patients (15.1%) experienced MACE, compared to 12 of 165 female patients (7.3%) (p < 0.0001). Clinical history showed a marked association with outcomes. Among patients with a highly suspicious history, 67.2% (exact number not stated) experienced MACE; by contrast, only 2.7% of the moderately suspicious group and 0% of the slight or non-suspicious group had events (p < 0.0001). These findings confirm that the HEART score’s predictive power is influenced by both objective factors (age, gender) and subjective clinical assessments (history of suspicion). Table 3 summarizes these associations, including p-values indicating the strength of statistical significance.

**Table 3:** Association of HEART score, gender, and clinical history with 30-day major adverse cardiac events (MACE) incidence Continuous variables are presented as mean ± standard deviation (mean ± SD) and were compared using the independent sample t-test. Categorical variables are shown as numbers and percentages (n (%)) and were analyzed using the chi-square test (χ²). Statistical significance was considered at p < 0.05. All reported p-values in this table were <0.0001, indicating highly significant associations.

Variable	Group	MACE incidence (n (%)) / mean ± SD	Test statistic	p-value
Mean HEART score	MACE vs. no MACE	6.54 ± 1.7 vs. 3.96 ± 2.0	t = 10.41	<0.0001
Gender	Male vs. female	36/239 (15.1%) vs. 12/165 (7.3%)	χ² = 8.62	<0.0001
History suspicion	Highly suspicious	67.2%	χ² = 120.91	<0.0001
	Moderately suspicious	2.7%		
	Slight/none	0%		

**Figure 2:**
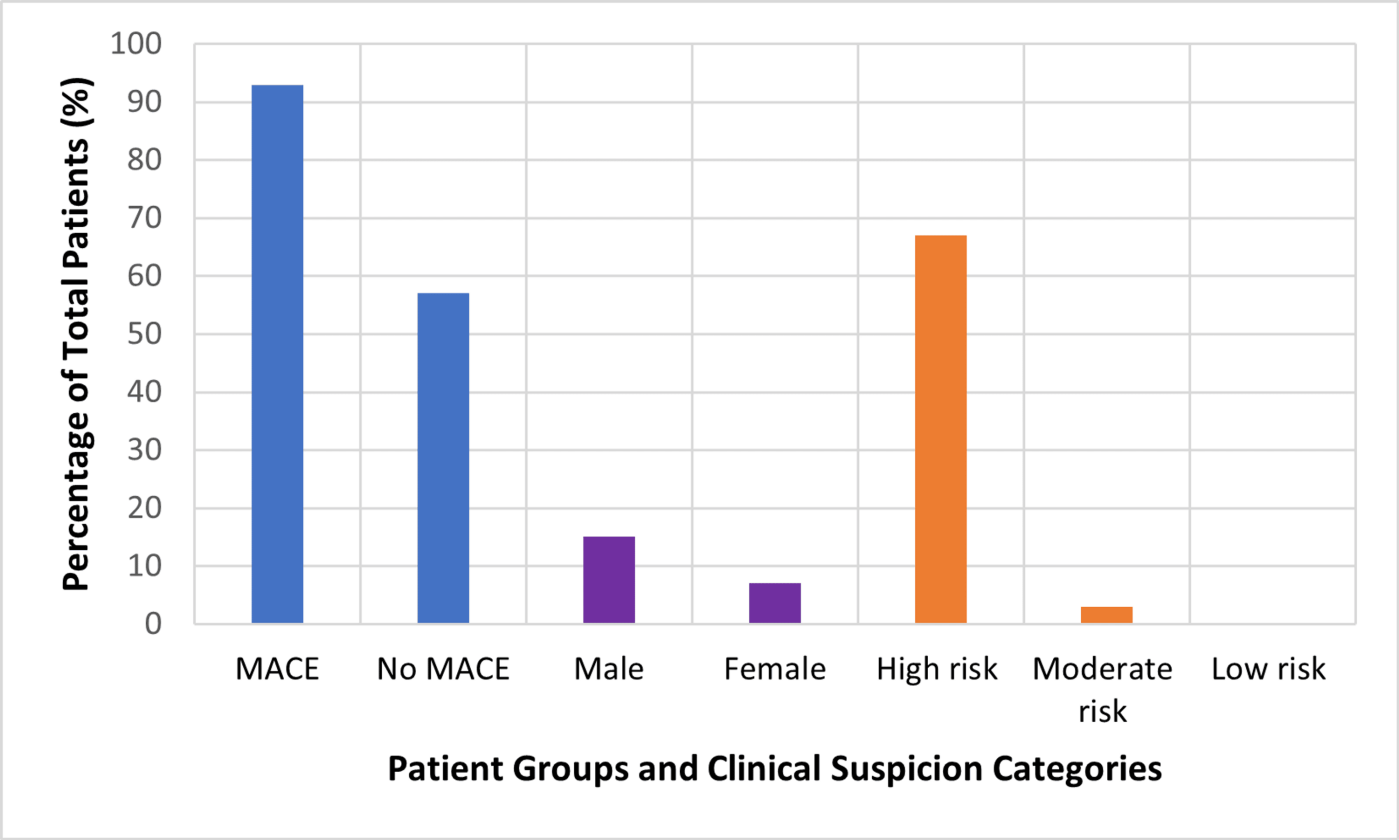
Distribution of patient characteristics and clinical suspicion categories

Figure 2 displays the percentage of the total study population (N = 404) across key clinical categories: patients who experienced MACE versus those who did not (no MACE), gender distribution (male and female), and clinical risk categories based on history assessment standardized as low risk, moderate risk, and high risk. Percentages reflect the proportion of the entire cohort within each category.

## Discussion

The research demonstrates that the HEART score is a reliable predictor of MACE within 30 days in patients presenting with chest pain. In our cohort, only three out of 325 patients (0.9%) in the low-risk group experienced MACE, indicating that 80.4% of patients were safely classified as low risk (HEART score 0-3) and unlikely to experience adverse outcomes. These findings align with prior studies, supporting the HEART score’s utility in guiding early discharge decisions in emergency settings.

Previous analyses have reported similar HEART score performance over longer follow-up periods, with minor variations due to differences in study design and populations [19-21]. Our 30-day follow-up highlights the tool’s value in high-acuity EDs, where rapid risk stratification is essential. By accurately identifying low-risk patients, the HEART score can potentially reduce unnecessary hospital admissions and invasive procedures, thereby optimizing healthcare resources. However, our study did not directly measure healthcare costs or iatrogenic complications, and these assertions are supported by prior literature rather than our data [22,23].

The study also emphasizes the importance of integrating demographic and clinical factors in risk assessment. Patients with a history classified as high risk exhibited substantially higher MACE rates (67.2%) compared to moderate (2.7%) and low suspicion groups (0%). This confirms that patient history is a meaningful component of the HEART score. Notably, sex differences were observed, with males experiencing higher MACE rates than females (15.1% vs. 7.3%, p < 0.0001). These differences may reflect biological factors such as plaque characteristics and behavioral variations in healthcare utilization [24].

While the HEART score performed consistently across sexes, the subjective nature of the history component introduces interobserver variability. To mitigate this, structured history-taking tools, standardized training programs, and decision aids should be implemented to enhance scoring consistency and reliability across providers [25]. In addition, our use of a point-of-care troponin I assay, although practical for emergency settings, may lack the sensitivity of high-sensitivity assays. Future incorporation of high-sensitivity troponin testing could improve early detection and HEART score accuracy [26].

Several limitations should be acknowledged. The single-center design and the relatively small number of high-risk patients (23/404) limit the generalizability of our findings. Multi-center studies with larger and more diverse cohorts are necessary to validate and expand upon our results [27]. While 98.3% of patients completed 30-day follow-up, exclusions due to incomplete outcome data may impact generalizability. Extending follow-up beyond 30 days may also clarify the long-term prognostic utility of the HEART score [28]. Future research could explore combining the HEART score with advanced imaging, biomarker panels, or artificial intelligence-based predictive tools to further enhance clinical utility [29,30].

In conclusion, the HEART score remains a validated, cost-effective, and clinically robust tool for triaging patients presenting with chest pain. It facilitates safe early discharge of low-risk patients, optimizes resource use, and supports targeted care delivery. While promising, its application to “real-world emergency settings” should be considered in the context of our study’s limitations, and ongoing multi-center validation is warranted to confirm broader applicability. Integration with emerging diagnostics and standardized clinical protocols may further enhance its impact in emergency medicine.

## Conclusions

This research helps fill a major gap in evidence by being among the first to demonstrate the utility of the HEART score in an Indian ED. Using data from 404 adults at a busy tertiary care hospital, the study shows that the HEART score can accurately predict 30-day MACE, with incidence rates of 89.2% in high-risk patients, 28.6% in moderate-risk patients, and only 0.9% in low-risk patients. While these findings strongly support the score’s risk stratification ability, it is important to acknowledge that further validation in larger and more diverse cohorts is needed before definitive conclusions can be drawn.

The study’s applicability is particularly relevant for EDs in LMICs, where resource constraints are common. It also highlights that factors such as age, sex, and clinical history influence risk categorization, suggesting potential for recalibrating the HEART score or developing personalized risk models incorporating these variables to improve accuracy. Despite limitations including the subjective nature of history assessment and use of conventional rather than high-sensitivity troponin assays, the findings indicate that the HEART score can enhance risk assessment, optimize resource use, and support clinical decisions. For broader clinical adoption, standardized clinician training, multi-center validation, and integration with local health policies are essential. Future studies with extended follow-up and external validation across diverse settings will further inform its generalizability and effectiveness.

## Appendices

Appendix A 

**Table 4:** Patient screening and exclusions in the chest pain cohort

Reason for exclusion	Number (n)	Percentage (%)
Total patients presenting with chest pain (screened)	450	100
Patients excluded due to non-cardiac chest pain	46	10.22
Patients enrolled in the study (cardiac chest pain)	404	89.78
Patients excluded due to incomplete HEART score (X)	4	0.99 (of 404)
Patients excluded due to incomplete follow-up (Y)	7	1.73 (of 404)
Patients included in cardiac risk analysis	393	97.29 (of 404)

Appendix B 

**Table 5:** Operational definition and scoring criteria used for HEART score calculation in this study ECG: electrocardiogram; CAD: coronary artery disease; DM: diabetes mellitus; HTN: hypertension; ng/mL: nanograms per milliliter

Component	Score = 0	Score = 1	Score = 2
History	Atypical chest pain, not suggestive of ischemia	Moderately suspicious history	Typical chest pain highly suggestive of ischemia
ECG	Normal ECG	Non-specific repolarization abnormalities	ST-segment depression or other ischemic changes
Age	<45 years	45–65 years	>65 years
Risk factors	No risk factors	1–2 risk factors (e.g., DM, HTN, smoking, etc.)	≥3 risk factors or known CAD
Troponin I	<0.08 ng/mL	0.08–0.24 ng/mL	>0.24 ng/mL

Appendix C 

**Table 6:** Stratified major adverse cardiac events (MACE) incidence by gender and clinical history suspicion categories

Subgroup	Number of patients (n)	MACE events (n)	MACE incidence (%)
Gender			
Male	239	36	15.1
Female	165	12	7.3
Clinical history suspicion			
Low suspicion	250	0	0.0
Moderate suspicion	110	3	2.7
High suspicion	44	30	67.2
